# On the geometric phases during radio frequency pulses with sine and cosine amplitude and frequency modulation

**DOI:** 10.1063/5.0138779

**Published:** 2023-08-07

**Authors:** Dennis J. Sorce, Shalom Michaeli

**Affiliations:** 1 Independent Researcher, 6 Stonegate Court, Cockeysville, Maryland 21030, USA; 2 Center for Magnetic Resonance Research, University of Minnesota, 2021 6th Street SE, Minneapolis, Minnesota 55455, USAs

## Abstract

In this work, we describe the formation of geometric phases during nonadiabatic frequency swept (FS) radio frequency (RF) pulses with sine amplitude modulation and cosine frequency modulation functions. The geometric phases during the FS pulse were analyzed using a Schrödinger equation formalism, and the unified analytical expression for the geometric phase was derived. We present the solutions for sub-geometric phase components incorporated in spinor wavefunctions for the RF Hamiltonian of spin ½ nuclei. We demonstrate that the geometric phases during sine/cosine RF pulses are opposite in signs for different initial conditions of the spinor and that geometric phases can accumulate in correspondence to different magnetization trajectories. The derived formalism could be extended for the evaluation of the geometric phases during a wide class of amplitude- and frequency-modulated pulses used in MRI and in high-resolution NMR.

## INTRODUCTION

The concept of phase is pervasive in nuclear magnetic resonance (NMR). It has been shown that if the Hamiltonian undergoes slow changes and returns to its initial state after adiabatic evolution, the system acquires a measurable phase of purely geometric origin, in addition to the well-known dynamic phase.^1,2^ The conceptualization of the geometric phase appeared initially in the works by Pancharatnam in polarization optics.^3^ Additionally, in his seminal contribution, Berry showed that the geometric properties of the system can define the phase that only appears during cyclic time variations of the Hamiltonian of the system and for adiabatic time traversals.^1,2^ This concept was later generalized by Aharonov and Anandan to the case where the time traversal does not need to be adiabatic and the state of the system only needed to be specified as cyclic.^4,5^ Observation of the geometric phase in biological systems, such as the brain and central nervous system, had been envisioned several decades ago.^6^ Recent work demonstrated the importance of the geometric phases for the description of molecular dynamics.^7^ However, direct observation of the geometric phase *in vivo* remains elusive because of the complexity of multiple relaxation pathways occurring in the living sample.

The geometric phase has unique features associated with the evolution of the quantum ensemble, and it is independent of the dynamic dephasing in the system. In contrast to the dynamic phase that is refocused in the Spin Echo (SE)-type experiment,^8,9^ the geometric phase depends on the geometry of the environment and could be added by combining and refocusing reverse frequency sweep using radio frequency (RF) pulses.^10,11^ Experimentally, several evolution circuits of magnetization had been used in MR for the detection of the geometric phases, and one of the most common is the cone circuit where in the tilted rotating frame of reference the angle varies between the axis of quantization of the laboratory and rotating frame.^12–14^

In NMR, the variations of B_0_ and B_1_ magnetic fields in the inhomogeneous sample cause different spin ensembles to acquire different dynamic phases, which could be refocused using spin echo and rotary echo strategies.^8,9,15^ It had been understood that when spin magnetization undergoes precession in the rotating frame, the geometric phase is formed with its sign defined by the direction of the frequency sweep, and generally, the cyclic evolution of magnetization results in phase accumulation between ±π.^11^ The geometric phase could be refocused along with the dynamic phases; however, it could be accumulated in the SE experiment by performing frequency sweep in opposite directions using frequency swept (FS) pulses.^11^ This procedure results in the cancellation of the dynamic phase while leading to two geometric terms that add up together.^11^ The cancellation of the dynamic phase using conventional SE approaches was utilized, such as for quantum computing by NMR^16^ and realization of a one-qubit quantum gates.^10^ For magnetic resonance applications, the refocusing could also be achieved by generating rotating frame rotary echoes when inverting the effective field halfway through the FS pulses.^17–19^ Although the concept of the geometric phases is well established in different disciplines and had been evaluated previously for NMR applications,^13,20^ to the best of our knowledge, the description of the geometric phase formation during FS pulses is still unavailable. Therefore, it is instrumental to evaluate how the geometric phase could be formed during the amplitude and frequency-modulated RF pulses operating in adiabatic and nonadiabatic regimes. This goal is motivated by the substantial merit of the FS pulses and their broad applicability for generating noninvasive MRI contrasts^21–25^ and for protein dynamic characterization in NMR.^26,27^

**FIG. 1.**
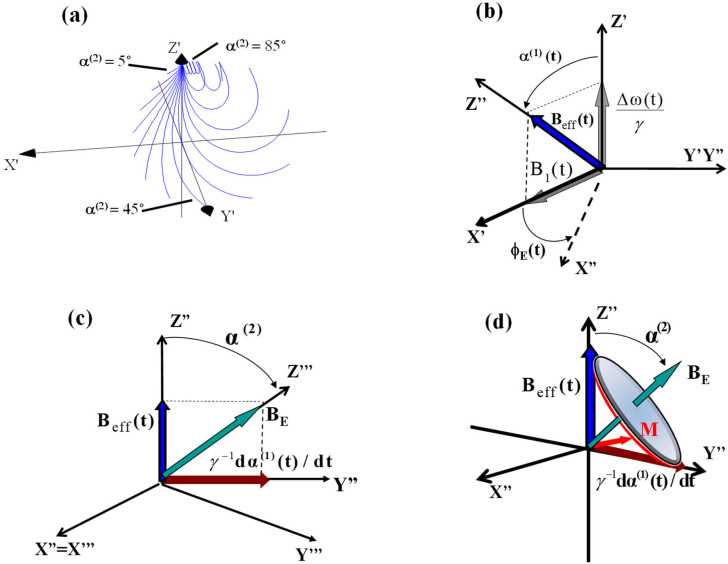
(a) Reproduced from the work of Liimatainen *et al.*^21^ This figure was published in the work of Liimatainen *et al.*, J. Magn. Reson. **209**, 269–276, Copyright Elsevier (2011). Magnetization trajectories during the sine/cosine RF pulse with different α^(2)^ angles. Fifteen α^(2)^ values were evenly distributed between 5° and 85°, and RF pulse amplitude and frequency modulation functions were generated with ω_1_^max^/(2π) = 625 Hz. The Runge–Kutta algorithm was used for simulating Bloch equations. Rotating frames of ranks n = 1 and n = 2 (b) and n = 2 and 3 (c). (b) The RF field **B**_**1**_**(t)** and frequency offset, Δω(t)/γ, are time-dependent functions in the laboratory frame of reference. The effective field **B**_eff_(t) is the vector sum of **B**_**1**_(t) and the fictitious component Δω/γ. The orientation between **B**_eff_(t) and the Z′ axis is described by an angle α^(1)^(*t*) that is time-variant. The second rotating frame (SRF) with the axis of quantization Z″ collinear to **B**_eff_(t) evolves in the first rotating frame (FRF). As a consequence of the time-dependence of α^(1)^(*t*), the FRF rotates around the Y′ axis leading to the SRF (n = 2). (c) The new effective field **B**_E_ is the vector sum of two field components: one of these components **B**_eff_(t) is the effective field in the FRF. The other component γ^−1^ dα^(1)^(t)/dt is the fictitious component that arises from the time-dependence of α^(1)^(t) the FRF and, thus, has an amplitude equal to γ^−1^dα^(1)^(t)/dt along the Y″ axis. ϕ_E_(t) indicates the phase of the transverse component of the RF field.^28,29^ (d) Schematic representation of magnetization precession in the SRF. Magnetization **M** undergoes precession around effective field **B**_E_ in the SRF as indicated by red arrows.^23^

Recently, a rotating frame relaxation method entitled Relaxation Along a Fictitious Field (RAFF) in the rotating frame of rank n (RAFFn) has been introduced.^21–23^ With RAFF2, the time-dependent Hamiltonian is transformed to a rotating frame of rank 2 resulting from nonadiabatic rotation of the effective field **B**_**eff**_ in the first rotating frame (FRF), which is the vector sum of **B**_**1**_(t) and the fictitious component Δω/γ (Fig. 1).

This rotation produces a fictitious field component (γ^−1^dα^(1)^/dt). The orientation between **B**_eff_(t) and the Z′ axis is described by an angle α^(1)^(t). The second rotating frame (SRF) with the axis of quantization Z″ collinear to **B**_eff_(t) evolves in the FRF. As a consequence of the time dependence of α^(1)^(t), the FRF rotates around the Y′ axis leading to the SRF (n = 2). The new effective field **B**_E_ is the vector sum of two field components: one of these components **B**_eff_(t) is the effective field in the FRF and the second is γ^−1^dα^(1)^/dt. We have shown that using different fictitious field angles α^(2)^ and amplitudes of the effective field, **B**_**E**_, in the SRF allowed us to generate novel MRI contrasts in the human brain.^21^ The formation of the geometric phases *in vivo*, although predicted in prior contributions, had not been quantified so far for FS pulses. Early work by Cui^30^ and subsequently by Lei and Zheng^31^ provided the analytical solution of the nonadiabatic geometric phases in the rotating systems. However, a detailed evaluation of the geometric phase formation for specific cases of the FS pulses operating in multiple rotating frames along with the experimental strategies for their detection in MR is not available.

In this work, we derived expressions for the geometric phases during the time-dependent RF Hamiltonian for spin ½. The RF pulses with sine amplitude modulation and cosine frequency modulation functions operating in the nonadiabatic regime were considered. We have utilized the formalism proposed by Suzuki *et al.*^28^ and first explicated by Messina *et al.*^29^ We elaborated on the dependence of the geometric phase on the initial conditions of the spinors $\left(\begin{array}{c} 1\\ 0 \end{array}\right)$ and $\left(\begin{array}{c} 0\\ 1 \end{array}\right)$ in the solution of the Schrödinger equation and evaluated geometric phases through their dependencies on the orientation of the effective field **B**_**E**_ in the SRF. Finally, we applied the developed formalism for the evaluation of the geometric phases during the RF pulse with sine amplitude modulation and cosine frequency modulation functions.

## THEORY

Conventionally, the time evolution of the quantum ensemble is described by the Schrödinger equation,

$$i\frac{\partial }{dt}\left|\Psi \left(t\right)\right\rangle =H\left(t\right)\left|\Psi \left(t\right)\right\rangle ,$$ (1)

with the wave function in the traditional adiabatic or nonadiabatic evolution representation written as follows:

$$|\Psi \left(t\right)〉=e^{i\overset{t}{\underset{0}{\int }}i\left\langle \Psi \left(t^{\prime }\right)|\frac{\partial }{\partial t^{\prime }}|\Psi \left(t^{\prime }\right)\right\rangle dt^{\prime }}e^{i\overset{t}{\underset{0}{\int }}\left\langle \Psi \left(t^{\prime }\right)|H\left(t^{\prime }\right)|\Psi \left(t^{\prime }\right)\right\rangle dt^{\prime }}|\Psi^{\prime }\left(t\right)〉.$$ (2)

The first exponential term in Eq. (2) represents the geometric phase, while the second exponential term is the dynamical phase. Here, |Ψ′(*t*)⟩ represents a general superposition of the eigenstates, and *H*(*t*) is the Hamiltonian. It is particularly appealing that the dynamic phase can be canceled by an appropriately chosen pulse sequence in MR, while the geometric component may survive. In accordance with the Centimeter–Gram–Second (CGS) system of units used in this paper, the units of the Hamiltonian in Eqs. (1) and (2) are in sec^-1^. A recent understanding of the origins of the geometric phases led to a conceptual advancement of nonadiabatic motion by introducing sub-geometric phases, thus unifying the adiabatic and nonadiabatic evolution of the Hamiltonian.^32^ The sub-geometric phase expression has a form equivalent to an adiabatic Berry phase,

$$G=\underset{i,j=1,2}{\sum }G_{i}^{j}=\underset{i,j}{\sum }\overset{t}{\underset{0}{\int }}\frac{i\left\langle \Psi_{i}^{j}\left(t^{\prime }\right)|\frac{d}{dt^{\prime }}|\Psi_{i}^{j}\left(t^{\prime }\right)\right\rangle }{\left\langle \Psi_{i}\left(t^{\prime }\right)|\Psi_{i}\left(t^{\prime }\right)\right\rangle }dt^{\prime }.$$ (3)

Here, the geometric phase G is the sum of the geometric phases of two possible initial conditions of the spinors $\left(\begin{array}{c} 1\\ 0 \end{array}\right)$ and $\left(\begin{array}{c} 0\\ 1 \end{array}\right)$ for the solution of the Schrödinger equation [Eq. (1)]. Since the total phase comprises both geometric and dynamic phases, the geometric phase can be evaluated as the difference between total and dynamic phases, as commonly exploited with spin echo sequences.^10,33^

In a series of papers appearing soon after Berry’s work, it had been demonstrated that the geometric phases can be determined through NMR experiments.^11–13,34,35^ A detailed description of the interacting ensemble was provided by Gamliel and Freed in the context of electron spin resonance (ESR).^36^ In this work, the conditions for experimental observations of the geometric phase by ESR were determined for the spin ensemble using the stochastic Liouville approach.^36^ Specifically, it had been shown that the experimental observation of Berry’s phases could become plausible for the spin ensemble undergoing slow evolution as compared to the rate of change of the Hamiltonian.

Here, the following generalized RF Hamiltonian for spin ½ nuclei is considered:

$$H^{RF2}\left(t\right)=\left(\begin{array}{cc} H_{11}\left(t\right) & H_{12}\left(t\right)\\ H_{21}\left(t\right) & H_{22}\left(t\right) \end{array}\right)=\left(\begin{array}{cc} \Delta \omega^{\left(1\right)}\left(t\right) & \omega_{1}^{\left(1\right)}\left(t\right)\\ \omega_{1}^{\left(1\right)}*\left(t\right) & -\Delta \omega^{\left(1\right)}\left(t\right) \end{array}\right),$$ (4)

where Δ*ω*^(1)^(*t*) and $\omega_{1}^{\left(1\right)}\left(t\right)$ are the elements of the Hamiltonian and “^∗^” indicates the complex conjugate. Corresponding to this Hamiltonian, there will be two associated eigenvectors.^28^ We note for the reader that the diagonal elements of Eq. (4) Hamiltonian are considered to be real numbers, while the off-diagonal elements could be complex and, in our case, real. We also indicate that the Hamiltonian is written in the second rotating frame with the axis X″Y″Z″ (Fig. 1).^37^ We define these associated eigenstates in terms of the time-evolution operator, *U*(*t*),

$$|\Psi_{1}\left(t\right)〉=U\left(t\right)\left(\begin{array}{c} 1\\ 0 \end{array}\right)\;\text{ and }\;|\Psi_{2}\left(t\right)〉=U\left(t\right)\left(\begin{array}{c} 0\\ 1 \end{array}\right),$$ (5)

where *U*(*t*) is given by^28^

$$U\left(t\right)=\left(\begin{array}{cc} U_{11}\left(t\right) & U_{12}\left(t\right)\\ U_{21}\left(t\right) & U_{22}\left(t\right) \end{array}\right).$$ (6)

The column vectors $\left(\begin{array}{c} 1\\ 0 \end{array}\right)$ and $\left(\begin{array}{c} 0\\ 1 \end{array}\right)$ are the chosen initial conditions for eigenvectors 1 and 2, respectively. Performing the matrix algebra in Eq. (5) using Eq. (6), we obtain

$$|\Psi_{1}\left(t\right)〉=\left(\begin{array}{c} U_{11}\left(t\right)\\ U_{21}\left(t\right) \end{array}\right)\;\text{ and }\;|\Psi_{2}\left(t\right)〉=\left(\begin{array}{c} U_{12}\left(t\right)\\ U_{22}\left(t\right) \end{array}\right).$$ (7)

Following the formalism by Susuki *et al.*^28^ and as first explicated by Messina *et al.*,^29^ we define the elements of the time evolution operator given in Eq. (6) as follows:

$$U_{11}\left(t\right)=Cos\left(\frac{\alpha^{\left(1\right)}\left(t\right)}{2}\right)Exp\left(-i\frac{\phi_{D}\left(t\right)}{2}\right),$$ (8a)

$$U_{22}\left(t\right)=Cos\left(\frac{\alpha^{\left(1\right)}\left(t\right)}{2}\right)Exp\left(i\frac{\phi_{D}\left(t\right)}{2}\right),$$ (8b)

$$U_{12}\left(t\right)=-i\;Sin\left(\frac{\alpha^{\left(1\right)}\left(t\right)}{2}\right)Exp\left(-i\frac{\phi_{O}\left(t\right)}{2}\right),$$ (8c)

$$U_{21}\left(t\right)=-i\;Sin\left(\frac{\alpha^{\left(1\right)}\left(t\right)}{2}\right)Exp\left(i\frac{\phi_{O}\left(t\right)}{2}\right).$$ (8d)

Here, *ϕ*_*D*_(*t*) = *ϕ*_*E*_(*t*) + *ϕ*(*t*), *ϕ*_*O*_(*t*) = *ϕ*_*E*_(*t*) − *ϕ*(*t*), orientation between *ω*_eff_(t) and the Z′ axis is defined by an angle *α*^(1)^(*t*), and *α*^(2)^(*t*) is the angle between the effective frequency *ω*_*E*_ generated in the SRF (or $\omega_{\text{eff}}^{\left(1\right)}$ frame) resulting from nonadiabatic rotation of $\omega_{\text{eff}}^{\left(1\right)}$, such as the effective frequency in the FRF, and Z″ (Fig. 1).^21,23^ By convention, the transformation from the Laboratory Frame (LF) to the FRF for rotating frame MRI is performed to eliminate the rapid precession rotation around the B_0_ field, and then, the subsequent transformation from the FRF to SRF is performed to deal with cases where there is time-evolution during the RF irradiation (Fig. 1).^22,37^

### Amplitude and frequency modulation functions

The basic relationships between RF amplitude, frequency offset, and effective frequency during the sine/cosine RF pulse are as follows:

$${\omega_{\mathrm{eff}}}^{\left(1\right)}\left(t\right)=\sqrt{{\omega_{1}}^{\left(1\right)}{\left(t\right)}^{2}+\Delta \omega^{\left(1\right)}{\left(t\right)}^{2}},$$ (9)

$$\alpha^{\left(1\right)}\left(t\right)=ArcTan\left(\frac{{\omega_{1}}^{\left(1\right)}\left(t\right)}{\Delta \omega^{\left(1\right)}\left(t\right)}\right),$$ (10)

where ${\omega_{1}}^{\left(1\right)}\left(t\right)=\gamma B_{1}\left(t\right)$ is the pulse amplitude, Δ*ω*^(1)^(*t*) is the time-dependent frequency offset, and *α*^(1)^(*t*) is the angle between quantization axis Z′ and the effective frequency $\omega_{\text{eff}}^{\left(1\right)}\left(\text{t}\right)\;=\;\gamma B_{\text{eff}}\left(\text{t}\right)$ in the FRF [Fig. 1(a)]. For the transformation to the SRF, we must define another angular quantity and another vectorial contribution to the effective field in the SRF [Fig. 1(b)]. To accomplish this, we write

$$\omega_{\text{eff}}^{\left(2\right)}\left(t\right)=\sqrt{\omega_{\text{eff}}^{\left(1\right)}{\left(t\right)}^{2}+{\left({\dot{\alpha }}^{\left(1\right)}\left(t\right)\right)}^{2}}$$ (11)

and

$$\alpha^{\left(2\right)}\left(t\right)=ArcTan\left(\frac{{\dot{\alpha }}^{\left(1\right)}\left(t\right)}{\omega_{\text{eff}}^{\left(2\right)}\left(t\right)}\right).$$ (12)

The angle for rotation around the effective frequency $\omega_{\text{eff}}^{\left(2\right)}\left(t\right)=\gamma B_{\text{E}}$ is given by

$$\phi_{\text{eff}}^{\left(2\right)}\left(t\right)=\overset{t}{\underset{0}{\int }}\omega_{\text{eff}}^{\left(2\right)}\left(t^{\prime }\right)dt^{\prime }.$$ (13)

The definition of specific forms for the RF modulation functions of the sine/cosine RF pulses operating in high rotating frames is given by Liimatainen *et al.*^21–23^ We have shown that the angle *α*^(2)^ is constant in the SRF for RF pulses with sine amplitude modulation with cosine frequency modulation functions. We thus define the amplitude and frequency modulation functions of the sine/cosine RF pulse with the orientation of the effective field **B**_**E**_ at *α*^(2)^(*t*) relatively to the Z″ axis as follows (Fig. 1):^21^

$${\omega_{1}}^{\left(1\right)}\left(t\right)=\omega_{1}^{\max}\;Sin\left(\omega_{1}^{\max}\tan\left(\alpha^{\left(2\right)}\right)t+\varphi_{0}\right),\text{0 }\leq t<T_{\text{p}}\text{/}2,$$ (14)

$$\Delta \omega^{\left(1\right)}\left(t\right)=\omega_{1}^{\max}\;Cos\left(\omega_{1}^{\max}\;\tan\left(\alpha^{\left(2\right)}\right)t+\varphi_{0}\right),\text{0 }\leq t<T_{\text{p}}\text{/}2.$$ (15)

Here, ${\omega_{1}}^{\max}$ is the maximum amplitude of the pulse. We arbitrarily choose *φ*_0_ = 0, and the phase modulation function can be obtained by integration of Eq. (15),

$$\phi \left(t\right)=\overset{t}{\underset{0}{\int }}\Delta \omega^{\left(1\right)}\left(t^{\prime }\right)dt^{\prime }=\frac{1}{\tan\left(\alpha^{\left(2\right)}\right)}Sin\left(\omega_{1}^{\max}\;\tan\left(\alpha^{\left(2\right)}\right)t\right).$$ (16)

With this pulse, the pulse frequency varies according to Eq. (15) and sweeps from its starting value toward the resonance condition with Δ*ω* = 0, while the pulse amplitude varies according to Eq. (14) in a sinusoidal manner. We consider cases when **M** is initially oriented along the Z″ axis, and the rotation of M is designed with **M** evolving on a cone with the precession defined by **B**_**E**_ and the angle *α*^(2)^. For the evolution time defined according to $T_{\text{p}}=\frac{4\pi }{\sqrt{2}\omega_{1}^{\max}}$, **M** undergoes a π/2 rotation and is oriented along the Y″ (or X″) axis at time T_p_/2. The inverse rotation of **M** can be created by instantaneously performing a π phase shift of **B**_**E**_ as given by the set of Eq. (17) as follows:

$$\omega_{1}^{\left(1\right)}\left(t\right)=\omega_{1}^{\max}\;Sin\left(\omega_{1}^{\max}\;\tan\left(\alpha^{\left(2\right)}\right)\left(t-\frac{T_{\text{p}}}{2}\right)+\pi \right)\text{,}T_{\text{p}}\text{/}2\leq t<T_{\text{p}}\text{,}$$ (17a)

$$\Delta \omega^{\left(1\right)}\left(t\right)=-\omega_{1}^{\max}\;Cos\left(\omega_{1}^{\max}\;\tan\left(\alpha^{\left(2\right)}\right)\left(t-\frac{T_{\text{p}}}{2}\right)\right)\text{,}T_{\text{p}}\text{/}2\leq t<T_{\text{p}}\text{,}$$ (17b)

$$\phi \left(t\right)=\frac{1}{\tan\left(\varepsilon \right)}\;\sin\left(\omega_{1}^{\max}\tan\left(\varepsilon \right)\left(t-\frac{T_{\text{p}}}{2}\right)\right)+\pi \text{,}T_{\text{p}}\text{/}2\leq t<T_{\text{p}}.$$ (17c)

Note that Eqs. (14) and (15) should be used in Eq. (4) of the Hamiltonian. Thus, following the definitions above for *ϕ*_*O*_(*t*) and *ϕ*_*D*_(*t*), the diagonal phases of the time-evolution operator in Eq. (8) are determined by *ϕ*_*D*_(*t*) and the off-diagonal elements are determined by *ϕ*_*O*_(*t*),

$$\phi_{O}\left(t\right)=\phi_{E}\left(t\right)-\phi \left(t\right)$$ (18a)

and

$$\phi_{D}\left(t\right)=\phi_{E}\left(t\right)+\phi \left(t\right).$$ (18b)

The time derivatives of *ϕ*(*t*) and *ϕ*_*E*_(*t*) are given by the following equations:

$$\;\frac{\dot{\phi }\left(t\right)}{2}=|\omega_{1}^{\left(1\right)}\left(t\right)|Sin\left(\phi_{E}\left(t\right)\right)\frac{1}{Sin\left(\alpha^{\left(1\right)}\left(t\right)\right)},$$ (19a)

$$\frac{{\dot{\phi }}_{E}\left(t\right)}{2}=\Delta \omega_{1}^{\left(1\right)}\left(t\right)-|\omega_{1}^{\left(1\right)}\left(t\right)|Sin\left(\phi_{E}\left(t\right)\right)\text{Cot}\left(\alpha^{\left(1\right)}\left(t\right)\right).$$ (19b)

Here, $\phi_{E}\left(t\right)=2\;Arc\text{Cot}\left(\pm 1-Csc\left(\alpha^{\left(1\right)}\left(t\right)\right)\right)$, and *ϕ*(*t*) is the phase, as derived by Suzuki *et al*.^28^ *ϕ*_*E*_(*t*) is used in Eq. (8) for the definition of the elements of the time evolution operator.

Next, following Eq. (5), we define

$$|\Psi_{1}\left(t\right)〉=\left(\begin{array}{c} Cos\left(\frac{\alpha^{\left(1\right)}\left(t\right)}{2}\right)Exp\left(-i\frac{\phi_{D}\left(t\right)}{2}\right)\\ -iSin\left(\frac{\alpha^{\left(1\right)}\left(t\right)}{2}\right)Exp\left(i\frac{\phi_{O}\left(t\right)}{2}\right) \end{array}\right)$$ (20a)

and

$$|\Psi_{2}\left(t\right)〉=\left(\begin{array}{c} -iSin\left(\frac{\alpha^{\left(1\right)}\left(t\right)}{2}\right)Exp\left(-i\frac{\phi_{O}\left(t\right)}{2}\right)\\ Cos\left(\frac{\alpha^{\left(1\right)}\left(t\right)}{2}\right)Exp\left(i\frac{\phi_{D}\left(t\right)}{2}\right) \end{array}\right).$$ (20b)

We then introduce the following definitions of the wave components:

$$|\Psi_{1}^{1}\left(t\right)〉=Cos\left(\frac{\alpha^{\left(1\right)}\left(t\right)}{2}\right)Exp\left(-i\frac{\phi_{D}\left(t\right)}{2}\right),$$ (21a)

$$|\Psi_{1}^{2}\left(t\right)〉=-i\;Sin\left(\frac{\alpha^{\left(1\right)}\left(t\right)}{2}\right)Exp\left(i\frac{\phi_{O}\left(t\right)}{2}\right),$$ (21b)

$$|\Psi_{2}^{1}\left(t\right)〉=-i\;Sin\left(\frac{\alpha^{\left(1\right)}\left(t\right)}{2}\right)Exp\left(-i\frac{\phi_{O}\left(t\right)}{2}\right),$$ (21c)

$$|\Psi_{2}^{2}\left(t\right)〉=Cos\left(\frac{\alpha^{\left(1\right)}\left(t\right)}{2}\right)Exp\left(i\frac{\phi_{D}\left(t\right)}{2}\right).$$ (21d)

Following the work of Wang *et al.*^32^ we define the sub-geometric phases as

$$G_{i}^{j}\left(t\right)=\overset{t}{\underset{0}{\int }}i\left\langle \Psi_{i}^{j}\left(\tau \right)|{\dot{\Psi }}_{i}^{j}\left(\tau \right)\right\rangle d\tau .$$ (22)

Here, $G_{i}^{j}\left(t\right)$ (*i*, *j* = 1, 2) is the definition of the sub-geometric phase component of the spinor solution of Schrödinger equations. Notably, the sum of sub-geometric phase components for the initial condition of spinors $\left(\begin{array}{c} 1\\ 0 \end{array}\right)$ and $\left(\begin{array}{c} 0\\ 1 \end{array}\right)$ is opposite in sign, namely, $G_{1}^{\mathrm{total}}\left(t\right)=-G_{2}^{\mathrm{total}};G_{1}^{1}\left(t\right)+G_{1}^{2}\left(t\right)=-\left(G_{2}^{1}\left(t\right)+G_{2}^{2}\left(t\right)\right)$. Based on our nomenclature, *G*_1_(*t*) corresponds to the initial condition of the spinor $\left(\begin{array}{c} 1\\ 0 \end{array}\right)$, and *G*_2_(*t*) indicates the initial condition of the spinor $\left(\begin{array}{c} 0\\ 1 \end{array}\right)$. The details of the derivations are given in Appendix A.

Using the same general concepts, we can also define sub-dynamic phases. For the convenience of the reader, we define total dynamic phases *D*_1_(*t*) and *D*_2_(*t*) for the initial conditions of the spinors $\left(\begin{array}{c} 1\\ 0 \end{array}\right)$ and $\left(\begin{array}{c} 0\\ 1 \end{array}\right)$, respectively, as follows:

$$D_{1}\left(t\right)=\overset{t}{\underset{0}{\int }}\left\langle \Psi_{1}\left(\tau \right)|H\left(\tau \right)|\Psi_{1}\left(\tau \right)\right\rangle d\tau ,$$ (23a)

$$D_{2}\left(t\right)=\overset{t}{\underset{0}{\int }}\left\langle \Psi_{2}\left(\tau \right)|H\left(\tau \right)|\Psi_{2}\left(\tau \right)\right\rangle d\tau ,$$ (23b)

with sub-dynamic phases given by

$$D_{i}^{j}\left(t\right)=\overset{t}{\underset{0}{\int }}\left\langle \Psi_{i}^{j}\left(\tau \right)|H_{ii}\left(\tau \right)|\Psi_{i}^{j}\left(\tau \right)\right\rangle d\tau .$$ (23c)

Here, *H*(*τ*) is defined to be a rank 2 matrix operator.^32^ To completely define sub-eigenfunctions, we operate in terms of a modified Schrödinger equation,

$$\frac{d|\Psi_{i}\left(t\right)〉}{dt}=-iH\left(t\right)|\Psi_{i}\left(t\right)〉,$$ (24)

where *i* corresponds to the initial conditions of the solutions of the Schrödinger equation and refers to the identity of the eigenfunction. Next, we use index *j* = 1, 2 to define the order for definition in the spinor column vector as follows:

$$|\Psi_{i}\left(t\right)〉=\left(\begin{array}{c} |\Psi_{i}^{1}\left(t\right)〉\\ |\Psi_{i}^{2}\left(t\right)〉 \end{array}\right)$$ (25)

or, for completeness,

$$|\Psi_{1}\left(t\right)〉=\left(\begin{array}{c} |\Psi_{1}^{1}\left(t\right)〉\\ |\Psi_{1}^{2}\left(t\right)〉 \end{array}\right)\;\text{ and }\;|\Psi_{2}\left(t\right)〉=\left(\begin{array}{c} |\Psi_{2}^{1}\left(t\right)〉\\ |\Psi_{2}^{2}\left(t\right)〉 \end{array}\right).$$ (26)

The Schrödinger equation is given by Eq. (24), with the Hamiltonian defined in Eq. (4). The general case for wave functions is given in Eq. (25), and the components of the wave functions are provided in Eq. (26). It should be noted that the imaginary part of the geometric phase was related to the dephasing of the geometric phase and its dissipation.^38^ Here, we are only focusing on the real part of the geometric phase during the sine/cosine Hamiltonian. Using Eqs. (1), (4), and (26), we write Eq. (27) as follows:

$$\begin{array}{c} i\frac{|\Psi_{i}^{1}\left(t\right)〉}{dt}=\Delta \omega^{\left(1\right)}\left(t\right)|\Psi_{i}^{1}\left(t\right)〉+\omega_{1}^{\left(1\right)}\left(t\right)|\Psi_{i}^{2}\left(t\right)〉,\\ i\frac{|\Psi_{i}^{2}\left(t\right)〉}{dt}={\omega_{1}}^{\left(1\right)}\left(t\right)|\Psi_{i}^{1}\left(t\right)〉-\Delta \omega^{\left(1\right)}\left(t\right)|\Psi_{i}^{2}\left(t\right)〉. \end{array}$$ (27)

Since Eq. (27) is a set of first-order differential equations in time, each spinor equation must have one initial condition specified for it. Using the NDSolve routine in Mathematica 12.2, we solve numerically the differential equation [Eq. (27)] with initial conditions given by Eq. (26) at *t* = 0.

We can then conveniently rewrite Eq. (27) in the following form that has the flexibility to be used with any chosen nomenclature for the two components of the spinor solutions of the Schrödinger equation,

$$\begin{array}{c} i\frac{da_{1}\left(t\right)}{dt}=\Delta \omega^{\left(1\right)}\left(t\right)a_{1}\left(t\right)+\omega_{1}^{\left(1\right)}\left(t\right)a_{2}\left(t\right),\\ i\frac{da_{2}\left(t\right)}{dt}={\omega_{1}}^{\left(1\right)}\left(t\right)a_{1}\left(t\right)-\Delta \omega^{\left(1\right)}\left(t\right)a_{2}\left(t\right), \end{array}$$ (28)

with initial conditions |*a*_1_(0) 〉= $\left(\begin{array}{c} 1\\ 0 \end{array}\right)$ and |*a*_1_(0) 〉= $\left(\begin{array}{c} 0\\ 1 \end{array}\right)$.

Next, we seek to incorporate the effects of sub-geometric and sub-dynamic phases in the definition of spinor wave function solutions. Hence, we define

$$|{\tilde{\Psi }}_{i}^{j}\left(t\right)〉=Exp\left(i\left(G_{i}^{j}\left(t\right)+D_{i}^{j}\left(t\right)\right)|\Psi_{i}^{j}\left(t\right)〉\right),$$ (29)

where

$$|\Psi_{i}^{j}\left(t\right)〉=Exp\left(i\beta_{i}^{j}\left(t\right)\right)|\phi_{i}^{j}\left(t\right)〉$$ (30)

and *j*, *i* = 1, 2. Based on Eqs. (29) and (30), we can write

$$|{\tilde{\Psi }}_{i}^{j}\left(t\right)〉=Exp\left(i\gamma_{i}^{j}\left(t\right)\right)|\phi_{i}^{j}\left(t\right)〉,$$ (31)

where

$$\gamma_{i}^{j}\left(t\right)=G_{i}^{j}\left(t\right)+D_{i}^{j}\left(t\right)+\beta_{i}^{j}\left(t\right).$$ (32)

Here, $\gamma_{i}^{j}\left(t\right)$ is a total phase, *i* is the spinor index, *j* is the sub-geometric phase index, and $\beta_{i}^{j}\left(t\right)$ are the phases of the spinor components as detailed below. More specifically,

$$|{\tilde{\Psi }}_{i}^{j}\left(t\right)〉=Exp\left(i\gamma_{i}^{j}\left(t\right)\right)|\phi_{i}^{j}\left(t\right)〉,$$ (33)

where the trigonometric components of the wave functions $|\phi_{i}^{j}\left(t\right)〉$ are given by

$$|\phi_{1}^{1}\left(t\right)〉=Cos\left(\frac{\alpha^{\left(1\right)}\left(t\right)}{2}\right),$$ (34a)

$$|\phi_{1}^{2}\left(t\right)〉=-i\;Sin\left(\frac{\alpha^{\left(1\right)}\left(t\right)}{2}\right),$$ (34b)

$$|\phi_{2}^{1}\left(t\right)〉=-i\;Sin\left(\frac{\alpha^{\left(1\right)}\left(t\right)}{2}\right),$$ (34c)

$$|\phi_{2}^{2}\left(t\right)〉=Cos\left(\frac{\alpha^{\left(1\right)}\left(t\right)}{2}\right).$$ (34d)

Next, we derive the expressions for the terms

$$\gamma_{i}^{j}\left(t\right),\text{ with }j,i=1,2,$$ (35)

as

$$\gamma_{1}^{1}\left(t\right)=G_{1}^{1}\left(t\right)+D_{1}^{1}\left(t\right)-\frac{\phi_{D}\left(t\right)}{2},$$ (36a)

$$\gamma_{1}^{2}\left(t\right)=G_{1}^{2}\left(t\right)+D_{1}^{2}\left(t\right)+\frac{\phi_{O}\left(t\right)}{2},$$ (36b)

$$\gamma_{2}^{1}\left(t\right)=G_{2}^{1}\left(t\right)+D_{2}^{1}\left(t\right)-\frac{\phi_{O}\left(t\right)}{2},$$ (36c)

$$\gamma_{2}^{2}\left(t\right)=G_{2}^{2}\left(t\right)+D_{2}^{2}\left(t\right)+\frac{\phi_{D}\left(t\right)}{2}.$$ (36d)

The solution for sub-geometric and sub-dynamic phases utilizing modified spinors can now be derived as

$$|{\tilde{\Psi }}_{1}\left(t\right)〉=\left(\begin{array}{c} Cos\left(\frac{\alpha^{\left(1\right)}\left(t\right)}{2}\right)Exp\left(i\gamma_{1}^{1}\right)\\ -iSin\left(\frac{\alpha^{\left(1\right)}\left(t\right)}{2}\right)Exp\left(i\gamma_{1}^{2}\right) \end{array}\right),$$ (37a)

$$|{\tilde{\Psi }}_{2}\left(t\right)〉=\left(\begin{array}{c} -i\;Sin\left(\frac{\alpha^{\left(1\right)}\left(t\right)}{2}\right)Exp\left(i\gamma_{2}^{1}\left(t\right)\right)\\ Cos\left(\frac{\alpha^{\left(1\right)}\left(t\right)}{2}\right)Exp\left(i\gamma_{2}^{2}\left(t\right)\right) \end{array}\right).$$ (37b)

Finally, the total geometric phase *G*_1_(*t*) is given by

$$G_{1}\left(t\right)=\overset{t}{\underset{0}{\int }}i\left\langle \Psi_{1}^{1}\left(\tau \right)|{\dot{\Psi }}_{1}^{1}\left(\tau \right)\right\rangle d\tau +\overset{t}{\underset{0}{\int }}i\left\langle \Psi_{1}^{2}\left(\tau \right)|{\dot{\Psi }}_{1}^{2}\left(\tau \right)\right\rangle d\tau .$$ (38)

Equations (39) and (40) are derived in greater detail in Appendixes A and B, respectively. Equation (39) describes the normalized geometric phase sub-component ${\bar{G}}_{1}^{1}\left(t\right)$ in relation to the tilt angle in the FRF *α*^(1)^(*t*) and the phase angle *ϕ*_*D*_(*t*) that is defined in Eq. (18),

$$G_{1}^{1}\left(t\right)=iLog\left(\frac{Cos\left(\frac{\alpha^{\left(1\right)}\left(t\right)}{2}\right)}{Cos\left(\frac{\alpha^{\left(1\right)}\left(0\right)}{2}\right)}\right)+\frac{1}{2}\phi_{D}\left(t\right).$$ (39)

Conversely, Eq. (40) relates the normalized geometric phase ${\bar{G}}_{1}\left(t\right)$ to the phase *ϕ*(*t*) using an integral relation involving the cosine of the tilt angle *α*^(1)^(*t*) and the time derivative of *ϕ*_*E*_(*t*),

$$G_{1}\left(t\right)=\frac{1}{2}\overset{t}{\underset{0}{\int }}\left({\dot{\phi }}_{E}\left(\tau \right)Cos\left(\alpha^{\left(1\right)}\left(\tau \right)\right)+\dot{\phi }\left(\tau \right)\right)d\tau .$$ (40)

Notably, Eq. (39) agrees algebraically and conceptually with Eq. (40). From Eqs. (39) and (40), it is possible to derive the real and imaginary parts of the sub-geometric components of ${\bar{G}}_{1}\left(t\right)$,

$$G_{1}^{1R}\left(t\right)=\frac{{\phi^{R}}_{E}\left(t\right)}{2}+\frac{\phi^{R}\left(t\right)}{2},$$ (41)

$$G_{1}^{1I}\left(t\right)=\frac{\phi^{I}\left(t\right)}{2}+Log\left(Cos\left(\frac{\alpha^{\left(1\right)}\left(t\right)}{2}\right)\right),$$ (42)

$$G_{1}^{2R}\left(t\right)=-\frac{{\phi^{R}}_{E}\left(t\right)}{2}+\frac{1}{2}\int_{0}^{t}Cos\left(\alpha^{\left(1\right)}\left(\tau \right)\right){{\dot{\phi }}^{R}}_{E}\left(\tau \right)d\tau ,$$ (43)

$$G_{1}^{2I}\left(t\right)=-Log\left(Cos\left(\frac{\alpha^{\left(1\right)}\left(t\right)}{2}\right)\right).$$ (44)

If *ϕ*(*t*) = *ϕ*^*R*^(*t*) + *iϕ*^*I*^(*t*), one can verify that

$$G_{1}\left(t\right)=\left(G_{1}^{1R}\left(t\right)+G_{1}^{2R}\left(t\right)\right)+i\left(G_{1}^{1^{I}}\left(t\right)+G_{1}^{2I}\left(t\right)\right),$$ (45)

where the left-hand side follows from Eq. (40) and the right-hand side is the result of Eqs. (41)–(44). This result lends support to the self-consistency between the derived relations in Eqs. (39) and (40). Appendix C shows that the final expressions for the geometric phase for the time-dependent angles *α*^(1)^(*t*) are given by

$$G_{1}\left(t^{\prime }\right)=\frac{1}{2}\left(\phi \left(t^{\prime }\right)+\overset{t^{\prime }}{\underset{0}{\int }}dt\;Cos\left.\left(\alpha^{\left(1\right)}\left(t\right)\right)\right.{\dot{\phi }}_{E}\left(t\right)\right),$$ (46)

and for stationary *α*^(1)^ cases,

$$G_{1}\left(t^{\prime }\right)=\frac{1}{2}\left(\phi_{D}\left(t^{\prime }\right)-\left(1-Cos\left(\alpha_{1}\right)\right)\phi_{E}\left(t^{\prime }\right)\right).$$ (47)

## RESULTS

**FIG. 2.**
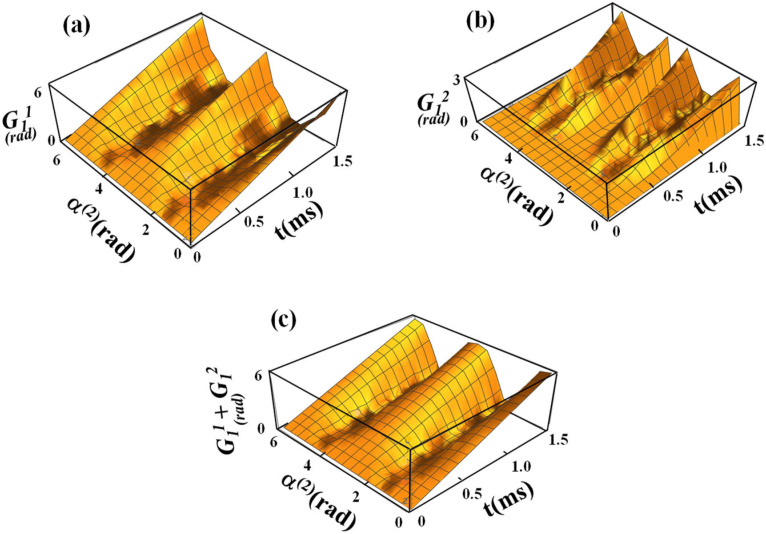
(a)–(c) Numerical calculations of sub-geometric phase components during the sine/cosine RF pulse with the initial condition $\left(\begin{array}{c} 1\\ 0 \end{array}\right)$ of the spinor as a function of α^(2)^(t) and time. For calculations of sub-geometric phase components, the propagators *U*_11_ and *U*_21_ were used according to Eq. (6). The sub-geometric phase components are shown in (a) and (b), and the sum of the sub-geometric phase components is displayed in (c). For calculations, ω_1_^max^/(2π) = 625 Hz was used.

**FIG. 3.**
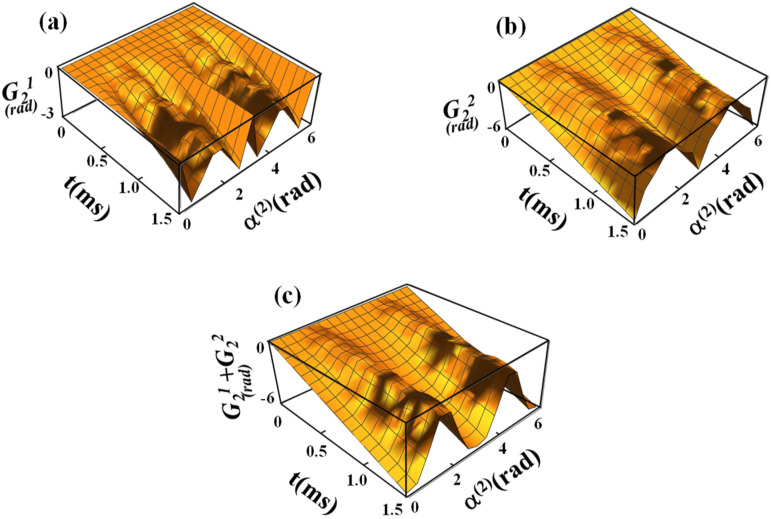
(a)–(c) Numerical calculations of sub-geometric phase components during the sine/cosine RF pulse with the initial condition $\left(\begin{array}{c} 0\\ 1 \end{array}\right)$ of the spinor as a function of α^(2)^(t) and time. For calculations of sub-geometric phase components, the propagators *U*_11_ and *U*_21_ were used according to Eq. (6). The sub-geometric phase components are shown in (a) and (b), and the sum of the sub-geometric phase components is displayed in (c). For calculations, ω_1_^max^/(2π) = 625 Hz was used.

Figure 1 illustrates the transformation of the spin ensemble from the FRF to the SRF. The nonadiabatic evolution of $\omega_{\text{eff}}^{\left(1\right)}\left(t\right)$ in the FRF leads to the generation of a fictitious field component γ^−1^dα^(1)^(t)/dt.^19^ Thus, the effective field that is formed in the SRF, **B**_**E**_, is the vector sum of two components, B_eff_(*t*) and γ^−1^dα^(1)^(t)/dt,^19^ and has different α^(2)^ relative to Z” of the SRF depending on B_eff_(*t*) and γ^−1^dα^(1)^(t)/dt. In Figs. 2 and 3, the calculations of sub-geometric phase components are shown for the spinor corresponding to initial conditions $\left(\begin{array}{c} 1\\ 0 \end{array}\right)$, $|\Psi_{1}^{1}\left(t\right)〉$, and $|\Psi_{1}^{2}\left(t\right)〉$ and $\left(\begin{array}{c} 0\\ 1 \end{array}\right)$, $|\Psi_{2}^{1}\left(t\right)〉$, and $|\Psi_{2}^{2}\left(t\right)〉$, respectively, using Eq. (22), namely, $G_{1}^{1}\left(t\right)=\overset{t}{\underset{0}{\int }}i\left\langle \Psi_{1}^{1}\left(\tau \right)|{\dot{\Psi }}_{1}^{1}\left(\tau \right)\right\rangle d\tau$ and $G_{1}^{2}\left(t\right)=\overset{t}{\underset{0}{\int }}i\left\langle \Psi_{1}^{2}\left(\tau \right)|{\dot{\Psi }}_{1}^{2}\left(\tau \right)\right\rangle d\tau$. The amplitudes of sub-geometric phases increase with the duration of the sine/cosine RF pulse and significantly depend on the angle between the effective field in the SRF and Z″, α^(2)^(t).

**FIG. 4.**
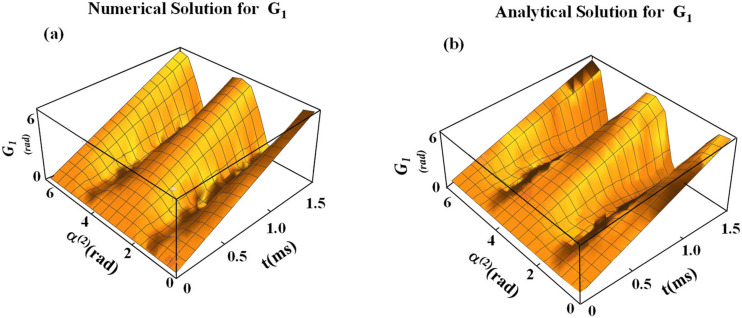
(a) and (b) Numerical and analytical calculation of the geometric phase during application of the sine/cosine RF pulse. Equation (46) was used for the analytical calculations. For calculations, ω_1_^max^/(2π) = 625 Hz was used.

In Fig. 4, we compare analytical [Eq. (40)] and numerical solutions [Eq. (27)] for the total geometric phase, which are in remarkable agreement. It should be noted that the dissipation of the geometric phase through the imaginary part was not explicitly included in the presented treatment, implying that the relaxation phenomena were not described.

**FIG. 5.**
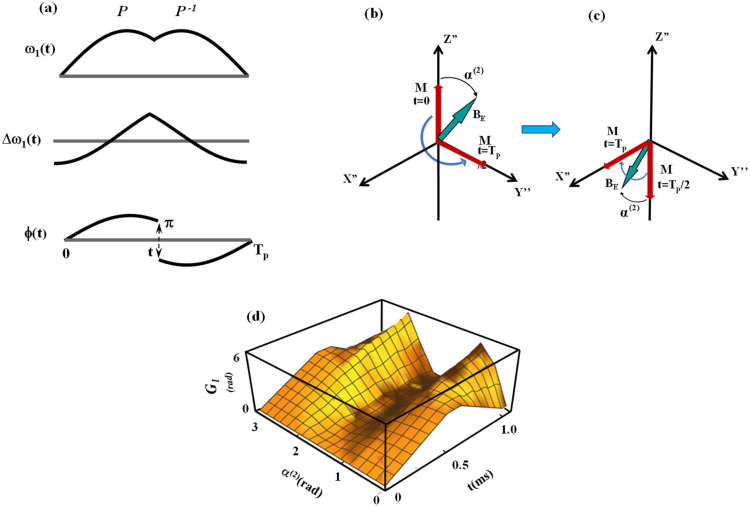
(a) Schematic representation of the amplitude, frequency, and phase modulation functions of the sine/cosine RF pulse for α^(2)^ = 45° used to design the evolution of **M** shown in (b) and (c). (b) Magnetization **M** (indicated in red) as viewed from the SRF undergoes rotation on a cone around **B**_**E**_ from the Z″ to Y″ axis during the first *P* segment. (c) During the *P*^−1^ segment, **M** evolves from −Z″ to X″. The modulation functions during *PP*^−1^ segments of the sine/cosine RF pulse are given in Eq. (16). Magnetization **M** evolves in different quadrants of the positive and negative hemispheres during the *P* and *P*^−1^ segments. (d) Numerical calculation of the geometric phase during the sine/cosine RF pulse shown in (a). The calculations were performed for the initial condition of the spinor $\left(\begin{array}{c} 1\\ 0 \end{array}\right)$. The evolution of magnetization **M** in the SRF is detailed in (b) and (c).

Figure 5 depicts one case of the RF pulses with sine amplitude modulation and cosine frequency modulation functions for α^(2)^ = 45° during *P* and *P*^−1^ segments, along with the magnetization path during the pulses. In Figs. 5(b) and 5(c), it is shown that magnetization **M** undergoes a rotation from the Z″ to Y″ axis of the SRF during the period [0, T_p_/2]. The rotation of **M** in the positive hemisphere is interrupted at the Y” axis, and **M** allows it to evolve in the negative hemisphere toward −Z″ during the period [T_p_/2, T_p_]. This rotation is achieved by instantaneously flipping **B**_**E**_ to π of the negative hemisphere, which is achieved by time-reversing both amplitude and phase and performing a π flip of the phase [Eq. (16)]. The pulse modulation functions are shown in Fig. 5(a). In Fig. 5(d), the numerical calculation of the geometric phase during the pulse waveforms represented in Fig. 5(a) is shown.

## DISCUSSION

In this work, we solved the Schrödinger equation for spinor components to obtain sub-geometric phases in the presence of a nonadiabatic RF Hamiltonian. We considered the RF pulse with sine amplitude modulation and cosine frequency modulation functions used in the recently developed rotating frame method called RAFFn.^21–23^ We provided an analytical representation of the propagators for spin ½ for the Hamiltonian that we defined in Eq. (4). This gives a closed form for the representation of the propagators in the FRF through α^(1)^, *ϕ*_*E*_, and *ϕ*(*t*). From two components of each spinor, we defined corresponding sub-geometric phases. For the general treatment, we solved the Schrödinger equation for different initial conditions of the spinors. Detailed analysis demonstrates that the geometric phases for initial conditions $\left(\begin{array}{c} 1\\ 0 \end{array}\right)$ and $\left(\begin{array}{c} 0\\ 1 \end{array}\right)$ are equal in magnitude but are opposite in their signs, which is in agreement with previously reported findings.^39^ We evaluated geometric phases via both analytical derivation and numerical solutions of the Schrödinger equation applied to the case of the nonadiabatic RF pulses operating in the rotating frame of rank n = 2. We derived the sub-geometric phase components (Fig. 2), and we determined through the evolution operator the functional relationship between the geometric phase and the RF pulse parameters that determine the magnetization path. The analytical and numerical solutions presented in this work are in remarkable agreement (Fig. 4).

During RF irradiation with sine/cosine RF pulses, the nonadiabatic rotation of the effective field in the FRF results in a fictitious field component, which leads to a formation of the SRF (rank n = 2).^21,23^ Figures 2 and 3 demonstrate the dependence of the geometric phase on α^(2)^ during the sine/cosine RF pulse. The calculations were performed for the initial condition of spinors $\left(\begin{array}{c} 1\\ 0 \end{array}\right)$ and $\left(\begin{array}{c} 0\\ 1 \end{array}\right)$. It can be seen that the geometric phases are formed predominantly for the angles α^(2)^ ∼ π/4 and α^(2)^ ∼ 3/4π with the maximal values accumulated at the end of the pulse. Conversely, no geometric phase formation was obtained for α^(2)^ ∼ π/2. In our previous work, the detailed analysis of the magnetization trajectories during the sine/cosine RF pulse was presented [Fig. 1(a)].^21^ For these analyses, the trajectories of the magnetization **M** in the FRF were calculated using the Runge–Kutta algorithm.^21^ The trajectories of **M** for a given α^(2)^ demonstrate that for small [such as α^(2)^ = 5°] and large [such as α^(2)^ = 85°s] angles, **M** nutates only slightly from the Z′ axis in the FRF. For intermediate values of α^(2)^, **M** nutates with larger angles and reaches the Y′ axis in the FRF for α^(2)^ = π/4. Notably, for the large angles α^(2)^, despite the high amplitude of the effective field **B**_**E**_ in the SRF, the nutation angle of **M** remains small because of fast oscillations of both amplitude and frequency modulations [see Eqs. (14), (15), and (17)]. The formation of the geometric phases during the sine/cosine RF pulse is closely related to the tip angles of **M** for various α^(2)^. Specifically, our results suggest that the maximal geometric phase is obtained for α^(2)^ ∼ π/4 and α^(2)^ ∼ 3/(4π), which corresponds to a maximal nutation of **M** in the FRF.

Previously, Jones *et al.*^11^ and subsequently Zhu and Wang^33^ have suggested that the geometric phase can be added in the SE experiment when the direction of the frequency sweep is reversed after the refocusing pulse, while it can be canceled along with the dynamic phase when the same frequency sweep is utilized. Such a strategy was also used in subsequent investigations.^10,39^ The geometric phase during sine/cosine RF pulses can be accumulated, such as by instantaneously reversing the effective field **B**_**E**_ in the SRF by π flip (Fig. 5). Magnetization evolves in the positive hemisphere during the first *P* segment of the RF pulses [Fig. 5(a)], but it evolves in different quadrants of the negative hemisphere during the *P*^−1^ segment [Figs. 5(b) and 5(c)]. The calculations of the geometric phase for the *PP*^−1^ segments shown in Fig. 5(a) are represented in Fig. 5(d). Despite **M** evolving in the negative hemisphere during the *P*^−1^ segment of the RF pulse, which is achieved by instantaneous π flip of the effective field **B**_**E**_, the accumulation of the geometric phase occurs because of the reverse frequency sweep. Such a strategy may offer an elegant solution for detecting the geometric phases in addition to the conventionally used spin echo-based approaches combined with reversed frequency sweeps.^10,11,16^

In this work, the effort of evaluating geometric phases during amplitude and frequency-modulated RF pulses was motivated by a possible contribution of the geometric phase to image contrast, which could be noninvasively generated during *in vivo* MRI. Our analytical and numerical evaluations set the basic framework for the description of the geometric phases during a wide class of amplitude and frequency-modulated RF pulses. It is likely that the geometric phases if inaccurately taken into consideration may bias the quantification of the relaxation rate constants during MRI pulse sequences. Finally, the development of novel techniques that allow for efficient refocusing of the dynamic phase while accumulating the geometric phase can open a new horizon for investigations of tissue microstructure and function.

## CONCLUSIONS

The formalism presented in this work can be used to describe the formation of geometric phases during RF swept pulses in MR operating in both adiabatic and nonadiabatic regimes. The formalism may be critical for accurately detailing noninvasive MRI tissue contrasts *in vivo* obtained during the application of RF waveforms.^22,23,40,41^ Since FS pulses are frequently used for protein dynamics characterization in high-resolution NMR, the formalism presented here could also be useful for relaxation dispersion analysis when FS pulses are utilized.^26,27,42^ To the best of our knowledge, a detailed evaluation of the relaxation processes with the inclusion of the effects of the geometric phases during amplitude- and frequency-modulated RF pulses has not been detailed in MR, and a limited effort had been dedicated to such a description.^36,43,44^ Initially, the description of the dissipative processes with the inclusion of the geometric phases was provided using the stochastic Liouville approach by Gamliel and Freed^36^ in Electron Paramagnetic Resonance (EPR). However, given significant differences between FS pulses operating in adiabatic and nonadiabatic regimes with substantial differences between modulation functions, a detailed description of the dissipation processes induced by geometric phases during FS pulses will require consideration in each case separately. Further investigations are thus warranted to properly consider the influence of the geometric phases on MRI contrasts generated noninvasively using FS pulses.

## Data Availability

All calculations included in this work were conducted using Mathematica 12 software package, and the programs/code can be provided by the authors upon request.

## APPENDIX A: DERIVATION OF NORMALIZED SUB-GEOMETRIC PHASE COMPONENT FOR THE SPINOR ELEMENT $|\Psi_{1}^{1}\left(t\right)〉$

We begin the derivation by writing the expression

$${\bar{G}}_{1}^{1}\left(t\right)=\overset{t}{\underset{0}{\int }}d\tau \frac{\left\langle \Psi_{1}^{1}\left(t\right)\|{\dot{\Psi }}_{1}^{1}\left(\tau \right)\right\rangle }{\left\langle \Psi_{1}^{1}\left(t\right)|\Psi_{1}^{1}\left(\tau \right)\right\rangle }.$$ (A1)

Using Eqs. (2) and (3) with a standard algebra, we obtain

$$\begin{array}{ll} |\overset{\cdot }{\Psi_{1}^{1}}\left(\tau \right)〉 & =-\left(Sin\left(\frac{\alpha^{\left(1\right)}\left(\tau \right)}{2}\right)\frac{\alpha^{\cdot \left(1\right)}\left(\tau \right)}{2}\right.\\ & \left.\;+i\;Cos\left(\frac{\alpha^{\left(1\right)}\left(\tau \right)}{2}\right)\frac{{\dot{\phi }}_{D}\left(t\right)}{2}\right)Exp\left(-i\frac{\phi_{D}\left(t\right)}{2}\right). \end{array}$$ (A2)

We then obtain the following equation by substituting Eq. (A2) into the integrand of Eq. (A1),

$$\frac{\left\langle \Psi_{1}^{1}\left(\tau \right)\|{\dot{\Psi }}_{1}^{1}\left(\tau \right)\right\rangle }{\left\langle \Psi_{1}^{1}\left(\tau \right)\|\Psi_{1}^{1}\left(\tau \right)\right\rangle }=\frac{-\left(Sin\left(\frac{\alpha^{\left(1\right)}\left(\tau \right)}{2}\right)Cos\left(\frac{\alpha^{\left(1\right)}\left(\tau \right)}{2}\right)\frac{\alpha^{\cdot \left(1\right)}\left(\tau \right)}{2}+i\;Cos{\left(\frac{\alpha^{\left(1\right)}\left(\tau \right)}{2}\right)}^{2}\frac{{\dot{\phi }}_{D}\left(t\right)}{2}\right)Exp\left(-i\left(\frac{\phi_{D}\left(t\right)}{2}-\frac{\phi {*}_{D}\left(t\right)}{2}\right)\right)}{Cos{\left(\frac{\alpha^{\left(1\right)}\left(\tau \right)}{2}\right)}^{2}Exp\left(-i\left(\frac{\phi_{D}\left(t\right)}{2}-\frac{\phi {*}_{D}\left(t\right)}{2}\right)\right)}.$$ (A3)

Simplifying the algebra, we obtain

$$\frac{\left\langle \Psi_{1}^{1}\left(\tau \right)\|{\dot{\Psi }}_{1}^{1}\left(\tau \right)\right\rangle }{\left\langle \Psi_{1}^{1}\left(\tau \right)\|\Psi_{1}^{1}\left(\tau \right)\right\rangle }=-\left(Tan\left(\frac{\alpha^{\left(1\right)}\left(\tau \right)}{2}\right)\frac{\alpha^{\cdot \left(1\right)}\left(\tau \right)}{2}+\frac{i}{2}{\dot{\phi }}_{D}\left(\tau \right)\right).$$ (A4)

Multiplying by *i*, we find

$$\left(-i\;Tan\left(\frac{\alpha^{\left(1\right)}\left(\tau \right)}{2}\right)\frac{\alpha^{\cdot \left(1\right)}\left(\tau \right)}{2}+\frac{1}{2}{\dot{\phi }}_{D}\left(\tau \right)\right).$$ (A5)

If we now integrate with respect to *τ*, we see that

$$\overset{t}{\underset{0}{\int }}\left(-i\;Tan\left(\frac{\alpha^{\left(1\right)}\left(\tau \right)}{2}\right)\frac{\alpha^{\cdot \left(1\right)}\left(\tau \right)}{2}d\tau \right)+\overset{t}{\underset{0}{\int }}\left(\frac{1}{2}{\dot{\phi }}_{D}\left(\tau \right)\right)d\tau$$ (A6)

becomes

$$\overset{t}{\underset{0}{\int }}\left(-i\;Tan\left(\frac{\alpha^{\left(1\right)}\left(\tau \right)}{2}\right)\frac{\alpha^{\cdot \left(1\right)}\left(\tau \right)}{2}d\tau \right)+\frac{1}{2}\phi_{D}\left(t\right).$$ (A7)

We next seek to evaluate the integral

$$\overset{t}{\underset{0}{\int }}\left(Tan\left(\frac{\alpha^{\left(1\right)}\left(\tau \right)}{2}\right)d\tau \right).$$ (A8)

Letting $\eta =\frac{\alpha^{\left(1\right)}\left(\tau \right)}{2}$, we obtain

$$2\overset{t}{\underset{0}{\int }}\left(Tan\left(\eta \right)d\eta \right).$$ (A9)

This can be rewritten as

$$-2\overset{t}{\underset{0}{\int }}\left(\frac{d\;Cos\left(\eta \right)}{Cos\left(\eta \right)}\right).$$ (A10)

Using standard calculus relationships, this becomes

$$-2\overset{t}{\underset{0}{\int }}d\;Log\left(Cos\left(\eta \right)\right)=-2\;Log\left(\frac{Cos\left(\eta \left(t\right)\right)}{Cos\left(\eta \left(0\right)\right)}\right).$$ (A11)

Substituting into Eq. (A7), it is found that

$$∠{\bar{G}}_{1}^{1}\left(t\right)=i\;Log\left(\frac{Cos\left(\frac{\alpha^{\left(1\right)}\left(t\right)}{2}\right)}{Cos\left(\frac{\alpha^{\left(1\right)}\left(0\right)}{2}\right)}\right)+\frac{1}{2}\phi_{D}\left(t\right)$$ (A12)

so that the real part of the normalized sub-geometric phase for the spinor component $|\Psi_{1}^{1}\left(t\right)〉$ is

$$\frac{\left(\phi_{E}\left(t\right)+\phi \left(t\right)\right)}{2}.$$ (A13)

## APPENDIX B: SYNOPSIS OF THE DERIVATION OF THE TOTAL NORMALIZED GEOMETRIC PHASE FOR THE INITIAL CONDITION $\left(\begin{array}{c} 1\\ 0 \end{array}\right)$ VS THE INITIAL CONDITION $\left(\begin{array}{c} 0\\ 1 \end{array}\right)$

We begin the derivation by writing accepted definitions of the normalized total geometric phases for the respective initial conditions (1 0) and (0 1), ${\bar{G}}_{1}\left(t\right)$ and ${\bar{G}}_{2}\left(t\right)$,^45–47^

$${\bar{G}}_{1}\left(t\right)=i\overset{t}{\underset{0}{\int }}\frac{\left\langle |\Psi_{1}\left(\tau \right)|{\dot{\Psi }}_{1}\left(\tau \right)\right\rangle d\tau }{\left\langle \Psi_{1}\left(\tau \right)|\Psi_{1}\left(\tau \right)\right\rangle },$$ (B1)

$${\bar{G}}_{2}\left(t\right)=i\overset{t}{\underset{0}{\int }}\frac{\left\langle \Psi_{2}\left(\tau \right)|{\dot{\Psi }}_{2}\left(\tau \right)\right\rangle d\tau }{\left\langle \Psi_{2}\left(\tau \right)|\Psi_{2}\left(\tau \right)\right\rangle }.$$ (B2)

We seek to prove that

$${\bar{G}}_{1}\left(t\right)=-{\bar{G}}_{2}\left(t\right).$$ (B3)

Given the definition of spinors given in Eq. (26) of the section titled Theory, we can write the following relations:

$$\left\langle \Psi_{1}\left(\tau \right)|{\dot{\Psi }}_{1}\left(\tau \right)\right\rangle =\left(\begin{array}{cc} \left\langle \Psi_{1}^{1}\left(\tau \right)\right| & \left\langle \Psi_{1}^{2}\left(\tau \right)\right| \end{array}\right)\left(\begin{array}{c} \left|{\dot{\Psi }}_{1}^{1}\left(\tau \right)\right\rangle \\ \left|{\dot{\Psi }}_{1}^{2}\left(\tau \right)\right\rangle \end{array}\right),$$ (B4)

which using the standard Dirac notation can be seen to equal

$$=\left\langle \Psi_{1}^{1}\left(\tau \right)|{\dot{\Psi }}_{1}^{1}\left(\tau \right)\right\rangle +\left\langle \Psi_{1}^{2}\left(\tau \right)|{\dot{\Psi }}_{1}^{2}\left(\tau \right)\right\rangle .$$ (B5)

Here, we note that the dot indicates taking the derivative with respect to *τ*.

Similarly, we can write

$$\left\langle \Psi_{2}\left(\tau \right)|{\dot{\Psi }}_{2}\left(\tau \right)\right\rangle =\left(\begin{array}{cc} \left\langle \Psi_{2}^{1}\left(\tau \right)\right| & \left\langle \Psi_{2}^{2}\left(\tau \right.\right| \end{array}\right)\left(\begin{array}{c} \left|{\dot{\Psi }}_{2}^{1}\left(\tau \right)\right\rangle \\ \left|{\dot{\Psi }}_{2}^{2}\left(\tau \right)\right\rangle \end{array}\right),$$ (B6)

which, then, is equal to

$$=\left\langle \Psi_{2}^{1}\left(\tau \right)|{\dot{\Psi }}_{2}^{1}\left(\tau \right)\right\rangle +\left\langle \Psi_{2}^{2}\left(\tau \right)|{\dot{\Psi }}_{2}^{2}\left(\tau \right)\right\rangle .$$ (B7)

Using the definitions in Eq. (7) of the text, we can then rewrite Eq. (B5) as

$$U_{11}\cdot \left(\tau \right){\dot{U}}_{11}\left(\tau \right)+U_{21}\cdot \left(\tau \right){\dot{U}}_{21}\left(\tau \right)$$ (B8)

and Eq. (B7) as

$$U_{22}\cdot \left(\tau \right){\dot{U}}_{22}\left(\tau \right)+U_{12}\cdot \left(\tau \right){\dot{U}}_{12}\left(\tau \right).$$ (B9)

For completeness, we write the following relationships, which easily follow from standard trigonometric identities:

$$\begin{array}{ll} \left\langle \Psi_{1}\left(\tau \right)|\Psi_{1}\left(\tau \right)\right\rangle & =\left\langle \Psi_{1}^{1}\left(\tau \right)|\Psi_{1}^{1}\left(\tau \right)\right\rangle +\left\langle \Psi_{1}^{2}\left(\tau \right)|\Psi_{1}^{2}\left(\tau \right)\right\rangle \\ & =|U_{11}\left(\tau \right){|}^{2}+|U_{21}\left(\tau \right){|}^{2}\\ & ={Cos}^{2}\left(\frac{\alpha^{\left(1\right)}\left(\tau \right)}{2}\right)+{Sin}^{2}\left(\frac{\alpha^{\left(1\right)}\left(\tau \right)}{2}\right)=1. \end{array}$$ (B10)

Similarly, for the (0 1) initial condition, we can state that

$$\begin{array}{ll} \left\langle \Psi_{2}\left(\tau \right)|\Psi_{2}\left(\tau \right)\right\rangle & =\left\langle \Psi_{2}^{1}\left(\tau \right)|\Psi_{2}^{1}\left(\tau \right)\right\rangle +\left\langle \Psi_{2}^{2}\left(\tau \right)|\Psi_{2}^{2}\left(\tau \right)\right\rangle \\ & =|U_{22}\left(\tau \right){|}^{2}+|U_{12}\left(\tau \right){|}^{2}\\ & ={Cos}^{2}\left(\frac{\alpha^{\left(1\right)}\left(\tau \right)}{2}\right)+{Sin}^{2}\left(\frac{\alpha^{\left(1\right)}\left(\tau \right)}{2}\right)=1. \end{array}$$ (B11)

Let us then compute

$$\left\langle \Psi_{1}\left(\tau \right)|{\dot{\Psi }}_{1}\left(\tau \right)\right\rangle \text{vs}\left\langle \Psi_{2}\left(\tau \right)|{\dot{\Psi }}_{2}\left(\tau \right)\right\rangle .$$ (B12)

Using the definitions of the matrix components of the time-evolution operators defined in Eq. (8) of the text,

$$\begin{array}{ll} & U_{11}\cdot \left(\tau \right){\dot{U}}_{11}\left(\tau \right)+U_{21}\cdot \left(\tau \right){\dot{U}}_{21}\left(\tau \right)\\ & \;=Cos\left(\frac{\alpha^{\left(1\right)}\left(\tau \right)}{2}\right)Exp\left(\frac{i}{2}\phi_{D}\left(\tau \right)\right)\\ & \;\;\times \frac{d\left(Cos\left(\frac{\alpha^{\left(1\right)}\left(\tau \right)}{2}\right)Exp\left(-\frac{i}{2}\phi_{D}\left(\tau \right)\right)\right)}{d\tau }\\ & \;\;+i\;Sin\left(\frac{\alpha^{\left(1\right)}\left(\tau \right)}{2}\right)Exp\left(-\frac{i}{2}\phi_{O}\left(\tau \right)\right)D_{\tau }\\ & \;\;\times \left(-i\;Sin\left(\frac{\alpha^{\left(1\right)}\left(\tau \right)}{2}\right)Exp\left(\frac{i}{2}\phi_{O}\left(\tau \right)\right)\right), \end{array}$$ (B13)

$$\begin{array}{ll} & U_{22}\cdot \left(\tau \right){\dot{U}}_{22}\left(\tau \right)+U_{12}\cdot \left(\tau \right){\dot{U}}_{12}\left(\tau \right)\\ & \;=Cos\left(\frac{\alpha^{\left(1\right)}\left(\tau \right)}{2}\right)Exp\left(-\frac{i}{2}\phi_{D}\left(\tau \right)\right)\\ & \;\;\times \frac{d\left(Cos\left(\frac{\alpha^{\left(1\right)}\left(\tau \right)}{2}\right)Exp\left(\frac{i}{2}\phi_{D}\left(\tau \right)\right)\right)}{d\tau }\\ & \;\;+i\;Sin\left(\frac{\alpha^{\left(1\right)}\left(\tau \right)}{2}\right)Exp\left(\frac{i}{2}\phi_{O}\left(\tau \right)\right)\\ & \;\;\times \frac{d\left(-i\;Sin\left(\frac{\alpha^{\left(1\right)}\left(\tau \right)}{2}\right)Exp\left(\frac{-i}{2}\phi_{O}\left(\tau \right)\right)\right)}{d\tau }. \end{array}$$ (B14)

By expanding Eq. (B13), one finds

$$\begin{array}{ll} & U_{11}\cdot \left(\tau \right){\dot{U}}_{11}\left(\tau \right)+U_{21}\cdot \left(\tau \right){\dot{U}}_{21}\left(\tau \right)\\ & \;=-\frac{1}{2}\left(Sin\left(\frac{\alpha^{\left(1\right)}\left(\tau \right)}{2}\right){\dot{\alpha }}^{\left(1\right)}\left(\tau \right)+i{\dot{\phi }}_{D}\left(\tau \right){Cos}^{2}\left(\frac{\alpha^{\left(1\right)}\left(\tau \right)}{2}\right)\right)\\ & \;+\frac{1}{2}\left(Sin\left(\frac{\alpha^{\left(1\right)}\left(\tau \right)}{2}\right){\dot{\alpha }}^{\left(1\right)}\left(\tau \right)+i{\dot{\phi }}_{O}\left(\tau \right){Sin}^{2}\left(\frac{\alpha^{\left(1\right)}\left(\tau \right)}{2}\right)\right), \end{array}$$ (B15)

which upon cancellation of terms and collection of variables leads to

$$\frac{i}{2}\left(-{\dot{\phi }}_{D}\left(\tau \right){Cos}^{2}\left(\frac{\alpha^{\left(1\right)}\left(\tau \right)}{2}\right)+{\dot{\phi }}_{O}\left(\tau \right){Sin}^{2}\left(\frac{\alpha^{\left(1\right)}\left(\tau \right)}{2}\right)\right),$$ (B16)

$$\begin{array}{ll} & U_{22}\cdot \left(\tau \right){\dot{U}}_{22}\left(\tau \right)+U_{12}\cdot \left(\tau \right){\dot{U}}_{12}\left(\tau \right)\\ & \;=-\frac{1}{2}\left(Sin\left(\frac{\alpha^{\left(1\right)}\left(\tau \right)}{2}\right){\dot{\alpha }}^{\left(1\right)}\left(\tau \right)+i{\dot{\phi }}_{D}\left(\tau \right){Cos}^{2}\left(\frac{\alpha^{\left(1\right)}\left(\tau \right)}{2}\right)\right)\\ & \;\;+\frac{1}{2}\left(Sin\left(\frac{\alpha^{\left(1\right)}\left(\tau \right)}{2}\right){\dot{\alpha }}^{\left(1\right)}\left(\tau \right)+i{\dot{\phi }}_{O}\left(\tau \right){Sin}^{2}\left(\frac{\alpha^{\left(1\right)}\left(\tau \right)}{2}\right)\right). \end{array}$$ (B17)

One then finds analogously that

$$\frac{i}{2}\left({\dot{\phi }}_{D}\left(\tau \right){Cos}^{2}\left(\frac{\alpha^{\left(1\right)}\left(\tau \right)}{2}\right)-{\dot{\phi }}_{O}\left(\tau \right){Sin}^{2}\left(\frac{\alpha^{\left(1\right)}\left(\tau \right)}{2}\right)\right).$$ (B18)

We call to the attention of the reader that, if one carefully reads Eqs. (B16) and (B18), they have opposite signs. To continue, we remind the reader that

$${\dot{\phi }}_{D}\left(\tau \right)={\dot{\phi }}_{E}\left(\tau \right)+\dot{\phi }\left(\tau \right)$$ (B19)

and that

$${\dot{\phi }}_{O}\left(\tau \right)={\dot{\phi }}_{E}\left(t\right)-\dot{\phi }\left(\tau \right).$$ (B20)

Upon substitution of Eqs. (B19) and (B20) into Eqs. (B16) and (B18) and using the trigonometric identities,

$${Cos}^{2}\left(\frac{\alpha^{\left(1\right)}\left(\tau \right)}{2}\right)-{Sin}^{2}\left(\frac{\alpha^{\left(1\right)}\left(\tau \right)}{2}\right)=Cos\left(\alpha^{\left(1\right)}\left(\tau \right)\right),$$ (B21)

$${Cos}^{2}\left(\frac{\alpha^{\left(1\right)}\left(\tau \right)}{2}\right)+{Sin}^{2}\left(\frac{\alpha^{\left(1\right)}\left(\tau \right)}{2}\right)=1,$$ (B22)

one finds that

$${\bar{G}}_{1}\left(t\right)=\frac{1}{2}\overset{t}{\underset{0}{\int }}\left({\dot{\phi }}_{E}\left(\tau \right)Cos\left(\alpha^{\left(1\right)}\left(\tau \right)\right)+\dot{\phi }\left(\tau \right)\right)d\tau ,$$ (B23)

$${\bar{G}}_{2}\left(t\right)=-\frac{1}{2}\overset{t}{\underset{0}{\int }}\left({\dot{\phi }}_{E}\left(\tau \right)Cos\left(\alpha^{\left(1\right)}\left(\tau \right)\right)+\dot{\phi }\left(\tau \right)\right)d\tau ,$$ (B24)

which leads to the expression

$${\bar{G}}_{1}\left(t\right)=-{\bar{G}}_{2}\left(t\right)$$ (B25)

as determined by numerical simulation (Fig. 3).

## APPENDIX C: DERIVATION OF ANALYTIC EXPRESSIONS FOR GEOMETRIC PHASE WITH TIME-DEPENDENT α^(1)^(t) AND α^(1)^ CONSTANT

We begin the derivation by writing

$$G_{1}\left[t^{\prime }\right]=i\overset{t^{\prime }}{\underset{0}{\int }}\left\langle \left.\Psi_{1}\left(t\right)\right|{\dot{\psi }}_{1}\left(t\right)\right\rangle dt.$$ (C1)

Without loss of generality, the normalization introduces a constant term and had been neglected.

We remind the reader that we follow the definitions above in Eq. (20),

$$|\Psi_{1}\left(t\right)〉=\left(\begin{array}{c} Cos\left(\frac{\alpha^{\left(1\right)}\left(t\right)}{2}\right)Exp\left(-\frac{i}{2}\phi_{D}\left(t\right)\right)\\ -i\;Sin\left(\frac{\alpha^{\left(1\right)}\left(t\right)}{2}\right)Exp\left(\frac{i}{2}\phi_{O}\left(t\right)\right) \end{array}\right),$$ (C2)

where as defined in the text

$$\phi_{D}\left(t\right)=\phi_{E}\left(t\right)+\phi \left(t\right),$$ (C3)

$$\phi_{O}\left(t\right)=\phi_{E}\left(t\right)-\phi \left(t\right).$$ (C4)

If one substitutes Eq. (C2) into Eq. (C1) and performs the necessary algebra, one obtains the following relation:

$${\hat{G}}_{1}\left(t\right)=\frac{-i}{2}\left({\dot{\phi }}_{D}\left(t\right){Cos}^{2}\left(\frac{\alpha^{\left(1\right)}\left(t\right)}{2}\right)-{\dot{\phi }}_{O}\left(t\right){Sin}^{2}\left(\frac{\alpha^{\left(1\right)}\left(t\right)}{2}\right)\right),$$ (C5)

where Eq. (C5) is the expression for ${\hat{G}}_{1}\left(t\right)$ before multiplication by *i* and the integration over *t*.

Let us consider two cases:

Case 1: *α*^(1)^(*t*) is time dependent.
Case 2: *α*^(1)^ is stationary.

Let us consider case 1: In Eq. (C6), we expand ${Sin}^{2}\left(\frac{\alpha^{\left(1\right)}\left(t\right)}{2}\right)=1-{Cos}^{2}\left(\frac{\alpha^{\left(1\right)}\left(t\right)}{2}\right)$ and collect terms in ${Cos}^{2}\left(\frac{\alpha^{\left(1\right)}\left(t\right)}{2}\right)$,

$${\hat{G}}_{1}\left(t\right)=\frac{-i}{2}\left(-{\dot{\phi }}_{O}\left(t\right)+{Cos}^{2}\left(\frac{\alpha^{\left(1\right)}\left(t\right)}{2}\right)\left({\dot{\phi }}_{D}\left(t\right)+{\dot{\phi }}_{O}\left(t\right)\right)\right).$$ (C6)

From Eqs. (C3) and (C4), we see that

$${\dot{\phi }}_{D}\left(t\right)+{\dot{\phi }}_{O}\left(t\right)=2{\dot{\phi }}_{E}\left(t\right).$$ (C7)

Let us expand using standard trigonometric identities,

$${Cos}^{2}\left(\frac{\alpha^{\left(1\right)}\left(t\right)}{2}\right)=\frac{\left(1+Cos\left(\alpha^{\left(1\right)}\left(t\right)\right)\right)}{2}.$$ (C8)

Substituting Eqs. (C7) and (C8) into Eq. (C6), we obtain

$${\hat{G}}_{1}\left(t\right)=\frac{-i}{2}\left(-{\dot{\phi }}_{O}\left(t\right)+\frac{\left(1+Cos\left(\alpha^{\left(1\right)}\left(t\right)\right)\right)}{2}{\dot{\phi }}_{E}\left(t\right)\right),$$ (C9)

$${\bar{G}}_{1}\left(t^{\prime }\right)=\frac{1}{2}\left(\phi \left(t^{\prime }\right)+\overset{t^{\prime }}{\underset{0}{\int }}dt\;Cos\left.\left(\alpha^{\left(1\right)}\left(t\right)\right)\right){\dot{\phi }}_{E}\left(t\right)\right).$$ (C10)

Here, we multiplied Eq. (C9) by *i* and integrated with respect to *t*. Equation (C10) is the final result, which is consistent with previous findings.^39^ It should be noted that in our nomenclature, ${\hat{G}}_{1}\left(t\right)$ and ${\bar{G}}_{1}\left(t^{\prime }\right)$ are the geometric phases before and after the integration, respectively.

For case 2, we consider *α*^(1)^ as stationary [see Eq. (10)] for time-invariant ω^(1)^ and Δω^(1)^, which could depend on constant amplitude and frequency offsets as was defined by Liimatainen *et al.* for the RAFF1 pulse.^22^ We make the substitution ${Cos}^{2}\left(\frac{\alpha^{\left(1\right)}\left(t\right)}{2}\right)=1-{Sin}^{2}\left(\frac{\alpha^{\left(1\right)}\left(t\right)}{2}\right)$ and collect terms in ${Sin}^{2}\left(\frac{\alpha^{\left(1\right)}\left(t\right)}{2}\right)$ to obtain

$${\hat{G}}_{1}\left(t\right)=\frac{-i}{2}\left({\dot{\phi }}_{D}\left(t\right)-{Sin}^{2}\left(\frac{\alpha^{\left(1\right)}\left(t\right)}{2}\right)\left({\dot{\phi }}_{D}\left(t\right)+{\dot{\phi }}_{O}\left(t\right)\right)\right).$$ (C11)

Once again, using Eqs. (C3) and (C4) and the identity

$${Sin}^{2}\left(\frac{\alpha_{1}\left(t\right)}{2}\right)=\frac{\left(1-Cos\left(\alpha^{\left(1\right)}\left(t\right)\right)\right)}{2},$$ (C12)

we obtain

$${\hat{G}}_{1}\left(t\right)=\frac{-i}{2}\left({\dot{\phi }}_{D}\left(t\right)-\frac{\left(1-Cos\left(\alpha^{\left(1\right)}\left(t\right)\right)\right)}{2}2{\dot{\phi }}_{E}\left(t\right)\right).$$ (C13)

Suppose we treat *α*^(1)^ as time-independent; then, multiplying Eq. (C14) by *i* and integrating with respect to t, we finally obtain the following equation:

$${\hat{G}}_{1}\left(t\right)=\frac{-i}{2}\left({\dot{\phi }}_{D}\left(t\right)-\left(1-Cos\left(\alpha^{\left(1\right)}\left(t\right)\right)\right){\dot{\phi }}_{E}\left(t\right)\right).$$ (C14)

The final solution for the stationary *α*^(1)^ is as follows:

$${\bar{G}}_{1}\left(t^{\prime }\right)=\frac{1}{2}\left(\phi_{D}\left(t^{\prime }\right)-\left(1-Cos\left(\alpha_{1}\right)\right)\phi_{E}\left(t^{\prime }\right)\right).$$ (C15)

This is the final form of the derived expression for case 2. It should be noted that case 2 directly follows from case 1 upon setting *α*^(1)^ as time-invariant.
